# Context-dependent roles of the gut microbiome in food allergy tolerance versus sensitization

**DOI:** 10.1080/19490976.2025.2590830

**Published:** 2025-12-02

**Authors:** Clara Delaroque, Mahesh S. Desai

**Affiliations:** a Department of Infection and Immunity, Luxembourg Institute of Health, Esch-sur-Alzette, Luxembourg

**Keywords:** Food allergy, gut microbiome, mucus, immunoglobulin A, immunoglobulin E, mucosal microbiome, diet

## Abstract

Exposure to food antigens that can trigger aberrant type-2 immunity is ubiquitous. However, only a subset of individuals develops allergy, implicating environmental drivers of sensitization, among which diet- and antibiotic-induced changes in intestinal microbiome activity stand out for their ability to alter host–microbe interactions at the gut mucosa. While efforts seek microbial signatures and microbiome-based therapies, the same microbes or pathways may foster either tolerance or sensitization depending on host and environmental context, which must be considered when designing interventions. We synthesize recent molecular insights into mucosal host–microbiome crosstalk, focusing on regulatory T cells, the colonic mucus barrier, and host immunoglobulins (IgA and IgE). Using examples of microbiome functional duality in which diet-driven altered microbial activities and secreted molecules such as lipopolysaccharides and flagellins yield opposing effects, we discuss the context-dependent mechanisms by which microbes either protect against or promote food allergy.

## Introduction

As the primary interface between the external environment and human tissues, the intestinal mucosa plays a crucial role in immune tolerance—the regulation of immune hyporesponsiveness to resident bacteria and dietary antigens—and is therefore essential for maintaining intestinal homeostasis.^1^^,^^2^ Disruptions in this delicate balance can lead to immune-related disorders; impaired tolerance toward commensal bacteria is associated with chronic intestinal inflammation, while loss of tolerance to food antigens underlies food allergies.^3^^,^^4^ Food allergies comprise several subtypes, classified according to their underlying immunological mechanisms. The most common form in Western populations is IgE-mediated food allergy, which affects approximately 1%–10% of the population worldwide, depending on age and geographical region.^5^ The process of pathogenesis involves immune responses in which IgE antibodies play a central role in triggering allergic reactions to specific food antigens,^3^^,^^6^ characterized by type 2 cytokines IL-4 and IL-13 signaling through IL4ra.^7-11^ In contrast, non-IgE-mediated food allergy—estimated to occur in 0.15%–0.7% of the population—is driven primarily by innate immune mechanisms that remain poorly understood.^3^^,^^5^

Identified risk factors for food allergy predominantly involve early-life disruptions of the gut microbiome, including mode of delivery, antibiotic use, and breastfeeding.^12^^,^^13^ Moreover, accumulating evidence shows that the microbiome from children with food allergy—or from corresponding mouse models—can induce allergy responses when transferred to previously-unaffected mice,^14-17^ whereas microbiome from healthy children can confer protection against food allergy development.^18^^,^^19^ These findings, together with evidence that the microbiome can modulate type 2 immunity,^20^ underscores the pivotal role of the gut microbiome in shaping susceptibility to food allergy, both through protective and deleterious effects.^14^^,^^16^^,^^18^

Over the recent decades, the prevalence of food allergy has risen sharply,^21-23^ particularly in industrialized countries,^5^^,^^24^ suggesting that lifestyle changes may contribute to disease development either directly or via microbiome-mediated pathways. Among environmental factors, diet has undergone substantial changes in recent decades.^25^^,^^26^ Modern diets are typically high in fat, sugar, and additives,^27^^,^^28^ while being deficient in dietary fiber.^26^^,^^28^^,^^29^ These dietary shifts profoundly influence the microbiome, not only in terms of composition but also in terms of functional capacity.^25^^,^^30^ Notably, fiber deprivation promotes the expansion of mucin-utilizing gut bacteria,^31-35^ compromising the integrity of the mucus layer that protects the colonic epithelium from direct contact with luminal bacteria. Interactions at the mucosal surface between the host and microbiome are critical for the development of immune tolerance, and dietary-mediated perturbation of this niche has been shown to promote inflammation and allergic response.^31-34^^,^^36^^,^^37^

In this review, we examine recent insights into the mechanisms underlying host–microbiome interactions at the mucosal surface and their role in initiating immune tolerance in the context of food allergy. We then focus on the dual role of specific microbial taxa and functions, highlighting how their effects on food allergy development can be either protective or detrimental depending on the context.

### Mucosal immune cells as key regulators of oral tolerance

The mucosal immune system—including effector T cells, innate lymphoid cells (ILCs), as well as myeloid cells—usually restrains inflammatory responses to dietary antigens and resident commensals. To prevent uncontrolled pathological immune activation, the intestinal immune system is tightly regulated by key mechanisms, among which CD4⁺ regulatory T (Treg) cells play a central role. These cells are characterized by constitutive CTLA-4, inducible T cell costimulator (ICOS), IL-10, TGF-*β*, and IL-35, suppressing myeloid cells and activating the bystander T cells to maintain immune tolerance to dietary antigens and intestinal microbiome.^38^

Studies investigating Treg cell depletion have reported that their absence results in spontaneous multiorgan autoimmunity and colitis.^39-43^ Conversely, transferring Tregs from sensitized to nonsensitized mice in a food allergy model effectively mitigated disease development,^44^ underscoring their essential role in balancing pro- and anti-inflammatory mechanisms to regulate both autoimmune reactions and immune responses to foreign antigen exposure at mucosal surfaces. These dual immunoregulatory functions are carried out by two distinct Treg populations: thymus-derived Treg (tTreg) cells, and peripherally induced Treg (pTreg) cells which differentiate within mucosal compartments.^45-47^ While tTregs mediate self-antigen tolerance, pTregs play a central role in maintaining tolerance towards foreign antigens at mucosal surfaces.^48^

Elegant work by Josefowicz and colleagues reported that selectively blocking the differentiation of pTreg cells in mice did not result in autoimmune nor heightened pro-inflammatory responses, but rather resulted in exacerbated Th2-mediated immune responses at mucosal sites, characterized by hallmarks of allergic inflammation and asthma, as well as alterations in the gut microbial communities.^4^ These results suggest that the regulation of allergic responses primarily depend on Tregs induced in the mucosal compartment, particularly within the intestinal mucosa.^4^^,^^49^ pTreg induction occurs through interactions between naïve T cells and antigen-presenting cells (APCs). While intestinal ILC3s are necessary and sufficient to promote microbiota-specific Tregs^50^ through a mechanism dependent on STING (stimulator of interferon genes) signaling,^51^ recent studies have also highlighted the central role of RORγt-expressing tolerogenic dendritic cells (tolDCs) in driving pTreg induction toward dietary antigens.^52^^,^^53^ In genetically modified mice, depletion of these cells abrogates pTreg induction, resulting in oral tolerance failure and development of low-grade intestinal inflammation.^52^^,^^53^ Mechanistic investigations have shown that tolDC-mediated tolerance induction depends on the transcription factors PRDM16 and RORγt, as well as Rorc(t) cis-regulatory element.^52^^,^^53^ Nevertheless, further studies are required to elucidate how these transcription factors regulate tolDC function in a way that specifically promote pTreg induction.

The induction of pTregs in the mucosal compartment is tightly regulated over time. For decades, medical recommendations have advised limiting children's exposure to well-known dietary allergens, such as peanuts. However, accumulating evidence now suggests that the early childhood period represents a critical window of opportunity for inducing mucosal tolerance to luminal allergens, including dietary antigens. The Learning Early About Peanut Allergy (LEAP) clinical trial, which investigated early peanut introduction in a population of high-risk infants, reported that early-life exposure to peanut allergens reduced the risk of peanut allergy at age 5 by 81%.^54^^,^^55^ This finding, along with others, not only revolutionized infant feeding guidelines worldwide but also reinvigorated scientific interest in oral tolerance induction as a fundamental immunological phenomenon. Interestingly, the effectiveness of early-life peanut exposure is highly age dependent: the greatest protection from allergy development is observed in younger children, while the effect diminishes when exposure occurs at later ages.^56^ Moreover, changes in microbial taxa and co-occurrence networks during the first year of life have been associated with atopic outcome in adulthood.^57^^,^^58^ Together, these findings suggest that the capacity to establish long-term tolerance to dietary antigens declines with age and is influenced by the microbiome.

This concept is further supported by cumulative evidence from mouse studies. Research in murine models has identified a critical early-life window during which naïve T cells exhibit a greater propensity to differentiate into pTregs within mucosal compartments in a microbiome-dependent manner.^13^^,^^40^^,^^59-61^ These early-life differentiated pTregs, in turn, provide long-lasting protection against exaggerated immune responses. However, when colonization or host–microbiome interactions are perturbed during this critical period—i.e., between birth and the transition to solid food at weaning—pTreg expansion fails to occur, thereby increasing susceptibility to disease development, including asthma and allergic diseases.^40^^,^^61-67^ For example, early-life antibiotic treatment during the preweaning period in mice was sufficient to drive lasting susceptibility to asthma^62^ and oxazolone-induced allergic reactions,^40^ accompanied by increased production of the type 2 immune response cytokines IL-4 and IL-13. This sustained susceptibility was mediated by impaired pTreg differentiation,^40^^,^^62^ as restoring the early-life microbiome could reverse disease susceptibility but not in mice with blocked Treg differentiation, highlighting the central role of Tregs.^40^ Further supporting this concept, associations have been observed between early-life environmental exposures and the development of food allergies in humans. For instance, children raised in farming environments are reported to be less susceptible to food allergies compared with those raised in urban settings,^68-71^ who exhibit reduced microbiota richness and an increase in inflammation-associated species.^71-73^

Additionally, while early-life microbiome and environmental factors are known to influence Treg populations, recent evidence suggests that other adaptive immune populations may also be durably imprinted. A specific Th2 effector cell population has been found to be elevated in both urban-raised and allergic children, suggesting a potential role in the development of food allergies.^74^ However, the mechanisms driving the expansion of these cells and their precise contribution to food allergy remain to be elucidated. These findings underscore the central role of the intestinal mucosal immune system and its tolerogenic properties in regulating immune responses during the onset of food allergy.

### Mucin–microbiome interactions in the modulation of food allergy

Extensive research in recent years has highlighted the crucial role of host–microbe interactions in the tolerogenic programming of the immune system. These interactions, and the associated molecular communications, occur predominantly within mucosal compartments, whose homeostasis has recently been identified as central to the development of food allergies.^1^ At the interface between intestinal-resident bacteria and the mucosal immune system in the lamina propria lies the intestinal epithelium and its overlying mucus layer.^75^^,^^76^ The intestinal mucus layer is primarily composed of mucins, a family of complex, glycosylated proteins. In the colon, the predominant mucin is characterized by a MUC2 protein core rich in serine and threonine residues that are extensively *O*-glycosylated.^76^^,^^77^ This mucus layer is organized into two distinct regions: an inner part that is impermeable to bacteria and an outer, more permeable part.^77^^,^^78^ It acts as a physical barrier that limits direct contact between microbes and host tissues, thereby reducing immune activation,^77-79^ and it delivers immunomodulatory signals to intestinal immune cells.^80^^,^^81^

**Figure 1.**
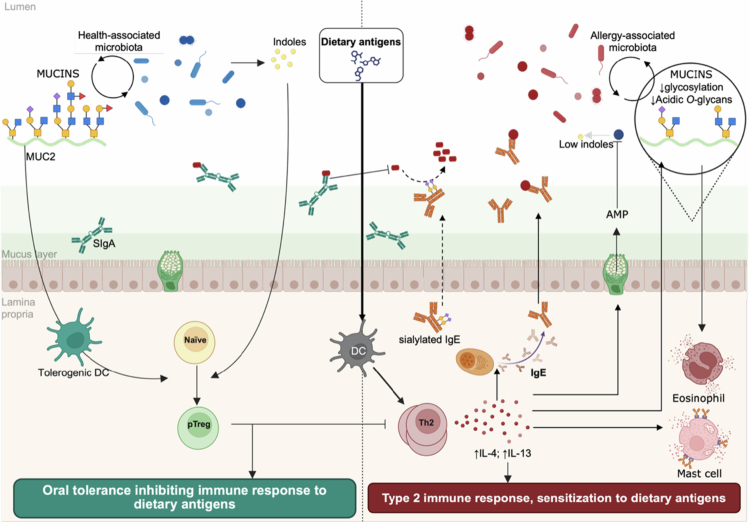
Host–microbiome interactions in the mucosal compartment modulate sensitization to dietary antigens. In healthy individuals, the colonic mucus layer supports beneficial host–microbiome interactions that promote the development and maintenance of immune tolerance. Microbiota members that penetrate the normally sterile inner mucus layer are neutralized by SIgA binding, thereby preventing inflammation. DCs receive tolerogenic signals from the mucus and, together with microbiome-derived signals—such as the microbial metabolite indole—promote immune tolerance through the differentiation of pTregs from naïve T cells, helping to prevent type 2 immune responses to dietary antigens. In contrast, disruption of host–microbiome interactions at the mucosal surface is commonly observed in patients with food allergies. This dysregulation includes reduced SIgA levels and increased translocation of IgE, which may alter microbiota composition and function. Additionally, allergy-associated cytokines IL-4 and IL-13 drive the expression of genes encoding antimicrobial proteins and promote changes in mucin *O*-glycosylation. Together, these alterations affect microbiota composition and function, potentially reinforcing allergic responses in a self-perpetuating feedback loop. Dashed arrows represent hypothetical mechanisms not yet supported by direct evidence; DC, dendritic cells; pTreg, peripherally differentiated regulatory T cells; SIgA, secretory immunoglobulin A; IgE, immunoglobulin E.

The colonic mucus layer has emerged as a key player in the establishment of oral tolerance.^1^ Studies in mucin-deficient mice have shown reduced mucosal Tregs and impaired oral tolerance.^80^^,^^82^ Notably, administration of mucus to these mice suppressed inflammatory responses and reduced allergy development.^80^ Moreover, exposure of eosinophils to mucus produced by cells stimulated with the allergy-related cytokine IL-13 resulted in enhanced degranulation,^81^ further supporting the immunomodulatory role of mucus depicted in Figure 1. Recent investigations into mucin glycosylation patterns have revealed that these modifications are dynamically regulated and closely linked to host health, particularly in the context of food allergy. In a mouse model of food allergy, the *O*-glycan profiles of intestinal mucins were compared between ovalbumin-sensitized and control mice.^83^^,^^84^ Sensitized mice exhibited a marked reduction in overall *O*-glycosylation, particularly in acidic *O*-glycans^83^^,^^84^ (Figure 1). These glycosylation changes coincided with shifts in the gut microbiota composition: sensitized mice showed increased abundances of the *Erysipelotrichaceae* and *Bacteroidaceae* families, while *Lachnospiraceae* and *Oscillospiraceae* families were reduced.^83^^,^^84^ These findings support accumulating evidence of a dynamic, bi-directional regulation between mucin structure and microbiome composition and function.^85^ Host factors also actively regulate mucin glycosylation. For example, ILC3s modulate colonic mucin galactosylation via IL-22, which in turn promotes expansion of the mucin-degrading commensal *Akkermansia muciniphila.*^86^ Similarly, ablation of genes encoding enzymes responsible for mucin sialylation leads to altered immune responses and changes in microbiota composition.^87^ Conversely, mucin structure is also shaped by the intestinal microbiome: microbiome transplantation into germ-free mice is sufficient to reproduce the donor's mucin glycosylation patterns,^88^ and supplementation with mucosal microbiota members such as *B. thetaiotaomicron* and *F. prausnitzii* modulates mucin production and glycosylation.^89^ Altogether, these studies highlight a dynamic interplay between the intestinal microbiome and mucins, both of which are involved in the development of food allergies. However, whether alterations in mucin glycosylation drive, result from, or occur independently of changes in the gut microbiota—and how these processes ultimately promote or protect against food allergy—remains open questions. A deeper understanding of these relationships may provide valuable insights into the mechanisms underlying disease development.

### Immunoglobulin–microbiome crosstalk in the mucus layer: role in food allergy

The mucus layer is enriched with immune molecules, including antimicrobial peptides (AMPs) and secreted immunoglobulins, which play a critical role in maintaining a safe distance between microbiota members and the epithelial surface by neutralizing bacteria capable of penetrating the mucus layer.^90^ Secretory IgA (SIgA) is the predominant secreted immunoglobulin in the colon and is primarily localized within the outer part of the colonic mucus layer, where it colocalizes with mucus-resident bacteria.^76^^,^^91^ Notably, a study involving patients with selective SIgA deficiency reports a prevalence of food allergy of approximately 25%, suggesting increased susceptibility^92^; however, these findings are based on a limited number of participants and should be interpreted cautiously. In mice, absence of IgA or the polymeric immunoglobulin receptor (pIgR)—which translocates IgA across the intestinal epithelium into the lumen—results in exaggerated immune responses to food antigens.^93^^,^^94^ This indicates effective IgA translocation into the intestinal lumen is essential for limiting the development of food allergies.^95^ Supporting this concept, multiple studies have reported reductions in fecal SIgA levels and the proportion of SIgA-bound bacteria in patients with allergic diseases^14^^,^^96-98^ (Figure 1). For example, recent investigation of SIgA-associated bacteria in fecal samples from children with IgE-mediated cow's milk allergy found not only a decreased proportion of IgA-bound bacteria, but also significant differences in the bacterial species targeted by SIgA compared with healthy controls.^97^ Specifically, SIgA binding to *Faecalibacterium* increased, while binding to *Ruminococcus* (from the *Ruminococcaceae* family) decreased, suggesting potential neutralization of health-associated species, such as *Faecalibacterium prausnizii*^99^ and a promotion of bacteria linked to allergic responses, such as *Ruminococcus gnavus.*^100^^,^^101^

The reduction in SIgA levels is typically accompanied by an increase in fecal IgE (Figure 1), which may contribute to the observed alterations in the gut microbiome of patients with IgE-mediated food allergies. Elevated fecal IgE levels have been documented in both murine models^34^ and human subjects,^14^^,^^102-104^ and inhibition of IgE translocation into the intestinal lumen has been reported to confer protection against allergic inflammation.^105^ Notably, we recently reported that this increase in luminal IgE can be modulated by both dietary factors and the microbiome.^34^ In a mouse model of food allergy colonized with a synthetic microbial community, animals maintained on a fiber-free diet exhibited heightened allergic responses and increased levels of fecal IgE-bound bacteria compared to those consuming a fiber-rich diet.^34^ Interestingly, removal of the mucin-degrading bacterium *A. muciniphila* from the microbial consortium markedly reduced the abundance of IgE-bound bacteria in mice fed the fiber-free diet.^34^ In contrast, under a fiber-rich diet, the presence of *A. muciniphila* was not associated with allergy development or increased IgE-bound bacteria.^34^ These findings suggest that these phenotypes may result from the specific activity of *A. muciniphila*, which is promoted under fiber-free conditions, as the responses of this mucin specialist could be different based on the dietary context.^32^ Although this work was shown only in mice, these findings broadly support the duality of the same microbes in either driving sensitization or protecting against it. *A. muciniphila* here only serves as a case in point, and it is possible that either *A. muciniphila* or bacteria other than *A. muciniphila* carry out similar dual roles in the human gut depending on the context. Given the well-established role of mucin degradation by *A. muciniphila* in shaping host health,^106-109^ together with our previous observation that fiber deprivation enhances its mucin-degrading activity,^31^ these findings also support the hypothesis that IgE production and/or its translocation into the intestinal lumen may be influenced by specific bacterial signals or mucin-degrading processes. Nevertheless, whether this influence is direct, indirect, or merely correlational remains to be clarified in future mechanistic studies, including investigations of other mucin-degrading bacteria.

Fecal IgE may actively contribute to shaping microbiome composition and function. This finding is supported by the differences in microbial profiles between individuals with IgE-mediated versus IgE-independent food allergies,^110^ which were further investigated using an IgE knock-in mouse model sensitized with ovalbumin.^111^ These mice exhibit exacerbated allergic responses to dietary antigens, increased abundances of *Lactobacillus* and *Butyricicoccus*, and concomitant reductions in *Clostridium* and *Lachnospiraceae* family members.^111^ These compositional changes are accompanied by functional alterations, including reduced concentrations of butyric acid and lysophospholipids,^111^ altogether suggesting that IgE may modulate microbiome composition and function.

While the precise mechanisms underlying IgE-mediated microbiome modulation remain to be fully elucidated, emerging findings suggest several hypotheses. For instance, IgE from individuals with peanut allergy demonstrates increased binding to fecal proteins, potentially indicating higher affinity for bacterial antigens compared with IgE from nonallergic controls.^104^ Such heightened affinity could influence mucosal microbial communities. Analogous to IgA, which selectively binds certain bacteria through interactions with glycans on the Fc region—a mechanism that contributes to the enrichment or exclusion of specific taxa^90^—IgE may similarly affect microbial dynamics. A recent study conducted an unbiased analysis of IgE glycosylation patterns in individuals with peanut allergy.^112^ They found an enrichment of sialic acid residues on circulating IgE compared to non-atopic individuals.^112^ Although this observation remains to be confirmed for luminally translocated IgE, sialic acid enrichment could serve as a nutrient source for specific microbial species, such as *R. gnavus*, which possesses the enzymatic machinery to cleave and metabolize sialic acids^113^ and has been reported to be enriched in food allergy patients.^100^^,^^101^

### Microbiome interaction with the immune components of the mucus layer and modulation of oral tolerance

Host immunoglobulins are not the only immune components involved in regulating microbial populations at the mucosal surface. Recent studies have highlighted the critical role of antimicrobial molecules—and their regulation by goblet cells—in fine-tuning allergic responses to dietary antigens.^17^^,^^114^^,^^115^ This concept was initially suggested in a study investigating the type 2 immune responses to parasitic infection in the small intestine, which induced the upregulation of the goblet cell-derived antimicrobial molecule resistin-like molecule-*β* (RELM-*β*) in an IL-13-dependent manner.^114^ This upregulation significantly impacted the mucosal microbiome, notably promoting expansion of *R. gnavus.*^114^ RELM-*β* was also identified as a key driver of colitis in *Muc2*^⁻/⁻^ mice, as knockdown of *Retnlb* (encoding RELM-*β*) significantly attenuated manifestations of colitis.^115^ These effects were mediated through modulation of the intestinal microbiome.^115^ Specifically, the pathogenic role of RELM-*β* in colitis was attributed to its antimicrobial activity against gram-positive *Lactobacillus* species, and supplementation with *Lactobacillus* spp. alleviated colitis in *Muc2*^⁻/⁻^ mice.^115^

A recent study by Stephen-Victor et al. reported that RELM-*β* expression is also upregulated in mice harboring a gain-of-function mutation in the IL-4 receptor alpha chain (*Il4ra*^*F709*^),^17^ a model for aberrant type 2 immune responses to dietary allergens. Increased RELM-*β* levels were similarly observed in children with food allergies compared to healthy controls.^17^ Mechanistic investigations revealed that RELM-*β* deficiency in *Il4ra*^*F709*^ mice was sufficient to abolish allergic reactions,^17^ and selective deletion of *Retnlb* in intestinal goblet cells conferred full protection, underscoring the central role of mucosal secretory cells in food allergy pathogenesis.^17^ RELM-*β* was also shown to act synergistically with IL-13 to drive the expression of other antimicrobial molecules, including SPPR2A, which further reduced *Lactobacillus* species due to their antimicrobial properties.^17^^,^^114^ The loss of these beneficial bacteria led to decreased fecal levels of indole, a tryptophan-derived microbial metabolite. Notably, similar reductions in indole levels have been observed in fecal samples from food-allergic infants.^17^ Indoles are crucial for the induction of RORγt⁺ pTregs via aryl hydrocarbon receptor (AhR) signaling,^20^^,^^116^and disruption of indole signaling has been reported to promote aberrant immune responses^117^^,^^118^ (Figure 1). Strikingly, supplementation of *Il4ra*^*F709*^ mice with indole-producing *Lactobacillus* strains was sufficient to protect against anaphylaxis, whereas treatment with genetically engineered strains unable to produce indole had no protective effect.^17^ These results are consistent with previous findings of *Lactobacillus*-associated protection against allergy development in mouse studies,^119-123^ as well as clinical interventions investigating the protective potential of *Lactobacillus* probiotics in patients with food allergy^124-127^. Collectively, these findings underscore the central role of bidirectional host–microbiome interactions at the intestinal mucosal interface in driving aberrant immune responses, leading to allergic diseases and systemic immune dysregulation.

### Context-dependent roles of the microbiome in food allergy: friend or foe?

Accumulating evidence link microbiome perturbations—particularly involving specific microbial taxa—to food allergies.^16^^,^^100^^,^^110^^,^^128^ However, whether these alterations are a consequence of aberrant allergic responses, a contributing factor to disease development, or both remains unclear. Multiple studies nevertheless support a causal role for microbiome modulation in the development of food allergies. Exacerbated allergic responses have been observed in germ-free mice, antibiotic-treated mice, and mice with early-life microbiome disruption.^40^^,^^62^^,^^117^^,^^129^^,^^130^ Moreover, transferring microbiome from food-allergic patients or allergic mouse models to allergy-prone mice intensifies allergic reactions,^14-17^ further suggesting causality. In addition, allergy-associated local inflammation, type 2 immune responses, increased luminal IgE with altered glycosylation, changes in mucin composition, and reduced luminal IgA may also drive shifts in microbial composition and function. Accordingly, recent work has sought to identify microbial taxa that either promote or alleviate food allergy. Rather than focusing on individual species, these studies highlight the importance of environmental influences on microbiome functions and metabolites that modulate host immune responses—such as indole. Supporting this concept, bacteriotherapy using *Clostridial* species confer protection against food allergy in mice.^14^ Notably, this protection was absent in *Myd88*-deficient mice, which lack responsiveness to most microbiome-derived signals,^14^^,^^118^underscoring the importance of microbiome-derived molecules in shaping type 2 immune responses.^131^

**Figure 2.**
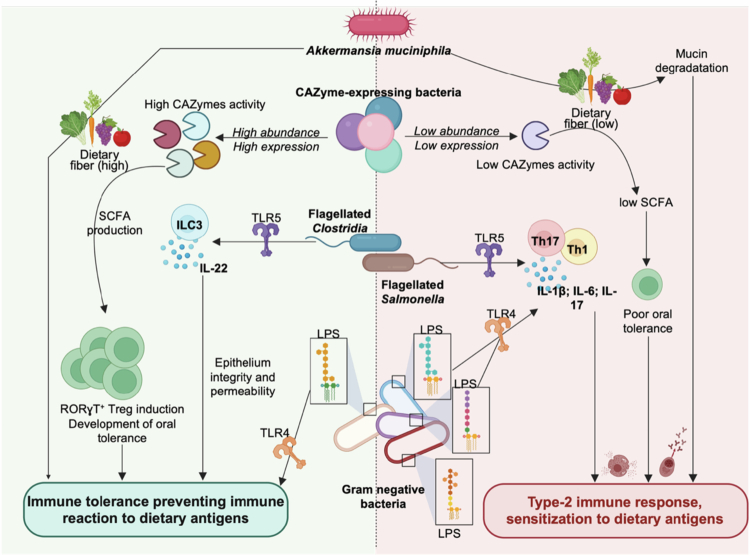
Context-dependent, microbiota-driven modulation of host sensitization to dietary antigens. Keystone members of the gut microbiome can exert dual effects on the host immune response to dietary antigens. For instance, *Akkermansia muciniphila* may shift toward mucin-degrading metabolism under conditions of limited dietary fiber, disrupting the mucosal barrier and promoting allergic responses. Similarly, the CAZyme profile of the microbiome influences oral tolerance, with reduced CAZyme abundance or expression being associated with impaired tolerance and increased susceptibility to allergy. Beyond microbial metabolism, microbiome-derived molecules also finely tune host responses to dietary antigens. Flagellin, for example, has been reported to either protect against or promote allergic responses depending on the bacterial origin, suggesting molecule-specific effects despite recognition by the same host receptor. LPS likewise displays context-dependent effects: low levels of LPS—or specific LPS structures from select bacterial species—are associated with protection from allergy, whereas elevated LPS levels, or LPS from proinflammatory species, can drive inflammation and promote type 2 immune responses upon exposure to dietary antigens. CAZyme, carbohydrate-active enzyme; LPS, lipopolysaccharide.

One such molecule is bacterial flagellin, which signals through host Toll-like receptor 5 (TLR5). Flagellin has been shown to synergize with AhR signaling to induce IL-22 production, thereby strengthening epithelial barrier integrity and protecting against food allergy.^118^ Interestingly, this effect was specific to *Clostridial* flagellin; in contrast, *Salmonella*-derived flagellin instead triggered a Th17-mediated type 3 immune response, despite engaging the same receptor^118^ (Figure 2). This dichotomy mirrors conflicting findings in the literature, where flagellins have been associated with both protective^132-134^ and detrimental^135^^,^^136^ roles in allergy. Strain-specific differences in flagellin structure and TLR5 interactions^137-141^ likely underlie these opposing outcomes. Indeed, recent work has reported that flagellin binding to TLR5 and downstream signaling varies widely across bacterial strains.^140^^,^^142^ These variations remain poorly understood and merit further study, particularly because flagellin–allergen fusion proteins are being explored as therapeutic candidates for food allergy.^143-145^

Other microbiome-derived molecules act in context-dependent ways, with lipopolysaccharide (LPS) as a prominent example. Metagenomic analyzes of patients with allergic diseases revealed a reduction in the expression of LPS biosynthesis genes, as confirmed by direct LPS quantification.^66^ Conversely, children with food allergies exhibit elevated levels of circulating LPS-binding protein^146^ (Figure 2). Mouse models further illustrate this duality: some studies report that LPS exposure worsens allergic reactions,^147^^,^^148^while others report that TLR4 deficiency (abolishing LPS sensing) exacerbates allergic responses,^149^^,^^150^ suggesting that LPS-TLR4 interaction may be involved in food allergy mitigation.^151^ These opposing effects may stem from strain-level structural differences or dosage effect, as low-dose LPS promotes Th2-type inflammation via TLR4, while high-dose exposure favors a type 1 response.^152^^,^^153^ This complexity, compounded by microbial dynamics, environmental influences, and host variability, complicates efforts to define consistent microbial markers of food allergy risk.

Importantly, bacteria influence immunity not only through inflammatory signaling but also via environmentally shaped, context-dependent interactions with the host. Cait et al. investigated such determinants by analyzing the fecal microbiome of atopic and healthy infants at 3 months and 1 year.^154^ While the overall microbiota composition was similar, approximately 40% of depleted taxa in atopic infants were species capable of fermenting complex carbohydrates.^154^ Functional metagenomics revealed a significant loss of carbohydrate-active enzymes (CAZymes) genes in the atopic group.^154^ These enzymes break down resistant starches into monosaccharides and disaccharides that serve as a cross-feeding substrates for short-chain fatty acid (SCFA)-producing bacteria. Because SCFAs promote Treg differentiation and oral tolerance,^40^^,^^155-158^ these findings suggest that disruption of microbial metabolic activity—through reduced CAZyme activity—may contribute to allergy development (Figure 2). Supporting this, high-fiber diet, which boost SCFAs production, protect mice against food allergy,^155^^,^^159^^,^^160^ but not in *Myd88*-deficient animals,^155^ highlighting the need for host–microbiota interactions signaling in effective immune regulation.

Adding to this complexity, our group and others have reported that the same bacterial species can exert divergent effects depending on environmental context.^161^ Similarly, bacteria typically associated with health benefits—such as *A. muciniphila*—may exert protective^106^^,^^107^^,^^162-164^ or detrimental^32^^,^^34^^,^^165^ effects depending on dietary context and host physiology. Environmental factors such as diet, maternal microbial transmission, and host immune status can profoundly alter microbial function and, in turn, shape allergic outcomes. For instance, maternal diet–induced changes in early-life microbiome composition have been shown to modulate offspring immunity.^13^^,^^67^^,^^166^ Specifically, while *A. muciniphila* has been reported to promote food allergy under conditions of fiber deprivation,^34^ its removal from the early-life microbial community has been associated with increased Th2 cell responses, which could possibly enhance susceptibility to food allergy in later life.^166^ These observations underscore that microbial function, rather than taxonomic identity alone, are central to determining host responses. Within this framework, the same organism can support tolerance in one setting yet exacerbate pathology in another, emphasizing the need to consider host–microbiome interactions within their broader ecological and environmental context.

## Conclusion

Although significant progresses have been made in understanding the role of the gut microbiome in food allergy, major knowledge gaps remain—particularly regarding how host–microbiome interactions influence individual's susceptibility, development, and severity of food allergies. While many studies have compared microbiota composition between food-allergic patients and healthy controls, emerging evidence suggests that environmental factors, such as diet, can modulate the effects of specific microbiota members or their derived compounds on the outcome of food allergy. This highlights the need to move beyond purely correlative studies towards mechanistic investigations, especially of host–microbiome interactions within the mucosal compartment, which are critical for the future development of effective therapeutic or preventive strategies. For instance, elevated levels of IgE have been observed in the feces of food-allergic patients in both mice and humans across multiple cohorts. However, how these molecules interact with the microbiome and the biological consequences of such interactions remain completely unknown. Furthermore, the impact of the microbiome on the host is likely highly individualized: certain bacteria or their derived molecules may exert either protective or harmful effects depending on the host's physiology and environmental context.^34^^,^^165^ Rather than focusing solely on specific taxa, increasing evidence points to the importance of understanding how microbial communities evolves over time and interact functionally. Innovative analytical approaches are now being employed to study these community-level dynamics and their associations with host health.^167^ Incorporating environmental variables into such analyzes could improve our ability to account for context-dependent effects, enhancing predictions of food allergy development, an approach already showing promising results,^168-170^ and enable the design of targeted, individualized, and environment-specific microbiome-based interventions.^26^
